# Supplementary figures and images for: A Weighted and Normalized Gould–Fernandez brokerage measure
Source: PLoS One. 2022 Sep 15;17(9):e0274475. doi: 10.1371/journal.pone.0274475 (PMC9477276; doi:10.1371/journal.pone.0274475)

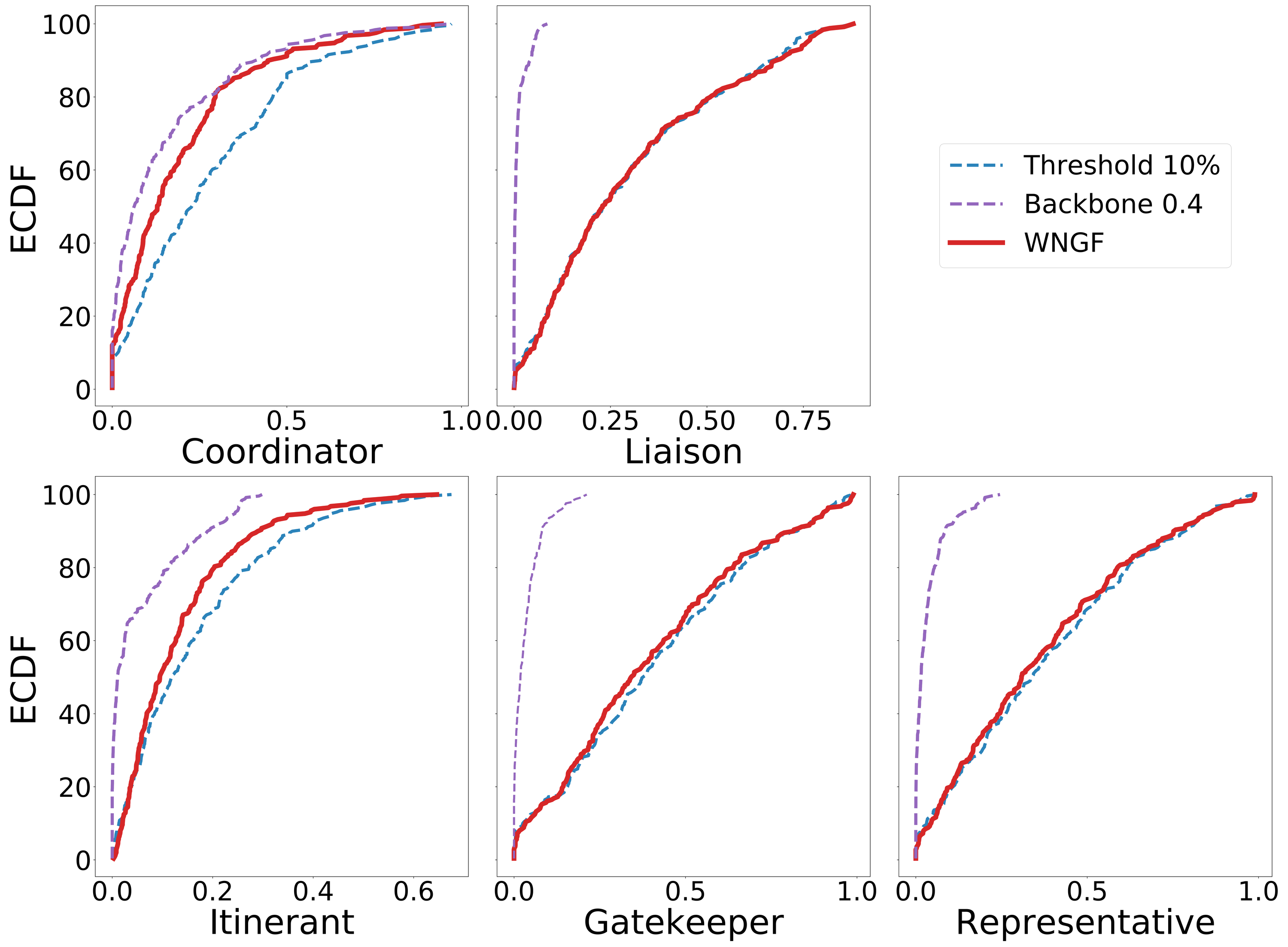

Supplement: S1 Fig — (TIF) [file pone.0274475.s001.tif]

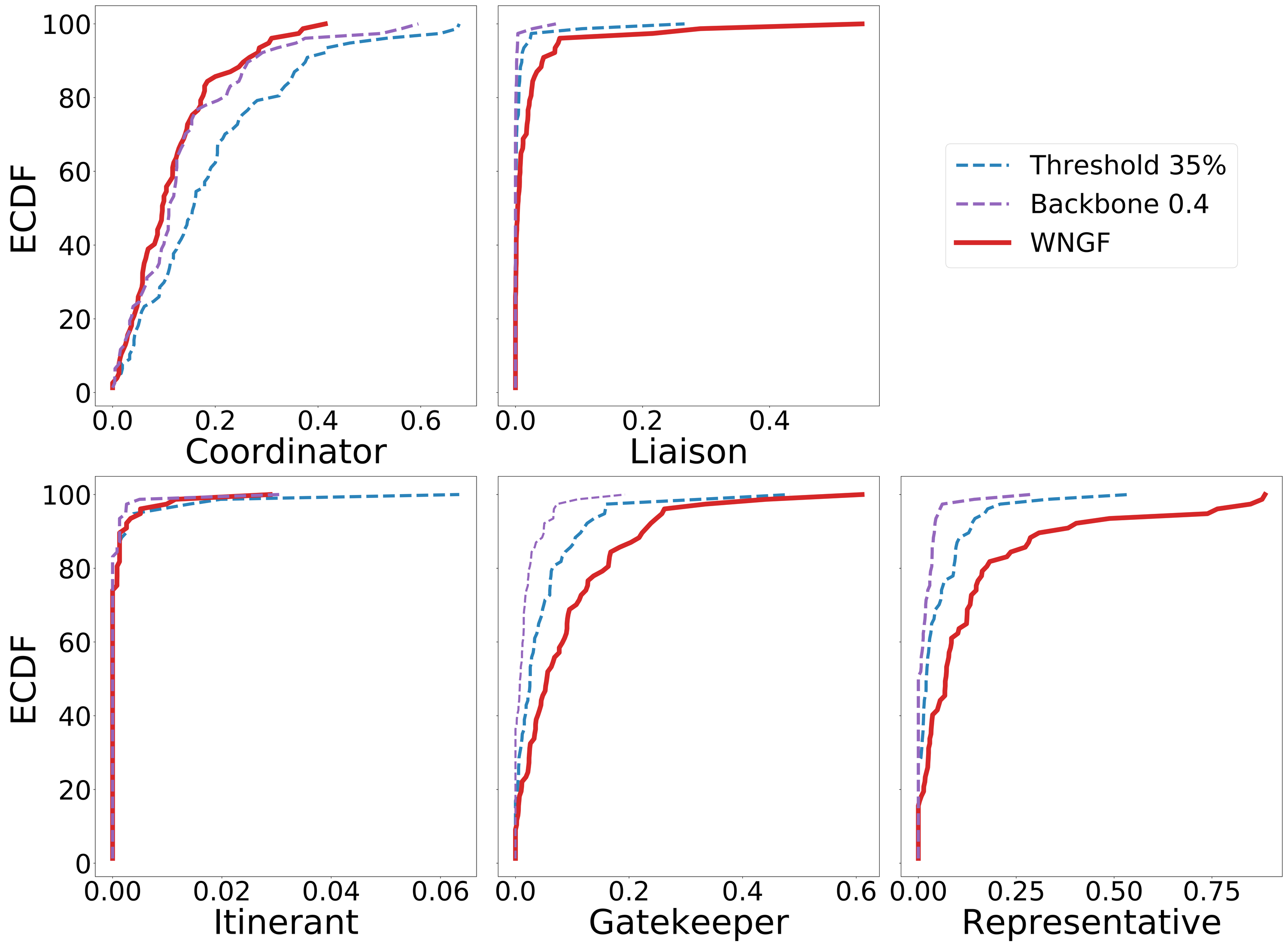

Supplement: S2 Fig — (TIF) [file pone.0274475.s002.tif]
