# Supplementary material for: A Weighted and Normalized Gould–Fernandez brokerage measure
Source: PLoS One. 2022 Sep 15;17(9):e0274475. doi: 10.1371/journal.pone.0274475 (PMC9477276; doi:10.1371/journal.pone.0274475)
Supplement: S1 Table — (DOCX) [file pone.0274475.s003.docx]

| EU countries with NUTS2 level data | EU countries with country level data | Non-EU countries and regions |
| --- | --- | --- |
| Austria | Cyprus | Australia and Oceania |
| Belgium | Bulgaria | Canada |
| Czech Republic | Romania | China |
| Denmark |  | India |
| Estonia |  | Indonesia |
| Finland |  | Japan |
| France |  | Korea |
| Germany |  | Middle and South America |
| Greece |  | Northern America |
| Hungary |  | Rest of the world |
| Ireland |  | Russia |
| Italy |  | Taiwan |
| Latvia |  | Turkey |
| Lithuania |  | United States |
| Luxembourg |  |  |
| Malta |  |  |
| Netherlands |  |  |
| Poland |  |  |
| Portugal |  |  |
| Slovakia |  |  |
| Slovenia |  |  |
| Spain |  |  |
| Sweden |  |  |
| United Kingdom |  |  |
