# Supplementary material for: A Weighted and Normalized Gould–Fernandez brokerage measure
Source: PLoS One. 2022 Sep 15;17(9):e0274475. doi: 10.1371/journal.pone.0274475 (PMC9477276; doi:10.1371/journal.pone.0274475)
Supplement: Attachment — Submitted filename: Response to Reviewers.pdf [file pone.0274475.s004.pdf]

Response to Reviewers

Reviewer #1

We would like to thank the reviewer for reading very carefully our work and for the insightful comments. They have helped us improve significantly the manuscript. (Comments by the reviewer are highlighted in blue)

Comment #1: The paper extend the Gould and Fernandez (GF) local brokerage measure to account for weighted edges (WNGF). I think the topic is interesting. The authors shows that: (i) WNGF generates valid results, similar to those generated by dichotomised measures, (ii) only through not-altering the network can one gain a perspective on the details of brokerage, (iii) since no alterations and decisions have to be made on how to dichotomise the network, the WNGF frees the user from the burden of making assumptions. I think that the paper deserve publication, even if I suggest to perform some minor revision in order to improve it.

We are glad to read that the reviewer found our paper deserves publication. We completely agree with the reviewer that some changes were indeed necessary to improve the paper. We will address these comments point-by-point.

Comment #2: Page 5: I think that some references to the percolation literature (and, in particular, to percolation centrality measures) could be a due enrichment of the literature review. Moreover, another paper that could be cited is this: https://doi.org/10.1093/icc/dtab078

We thank the reviewer for the suggestion on enriching the literature review. We have implemented this in the manuscript.

Comment #3: Page 13: the authors normalize the metrics, but they explain too briefly why it is relevant. For instance, I can think that a non-normalized metric could be better because a broker (for instance a gatekeeper) in a large group could be more relevant than a broker in a small group. Please improve the comments in this part.

We thank the reviewer for pointing out that further explanation was necessary, we have extended the justification. While we appreciate that in some cases a non-normalized metric might be preferable, our main reason for normalization is to generate comparable results. Furthermore, since GF brokerage identifies various types of brokers in different ways, the non-normalized measure would bias the results due to varying group sizes across brokerage types.

Comment #4: Page 17: the fact that WNGF output is almost equal to the “threshold” method output seems to reduce the relevance of the WNGF. However, on the other hand, the fact that they are so similar can strengthen the reliability of WNGF and “threshold” and can imply that “threshold” method outperforms “backbone” one.

We thank the reviewer for the insightful comment. This is a key point. The fact that the two methods bring consistent results is reassuring. In the paper we argue that both methods are valid. However, our method has three additional advantages: 1. information retention; 2. It uses data *as it is*, freeing the researcher from assumptions and discretionary choices which may be difficult to justify; 3. It allows for more granularity when the role of less connected nodes is considered. In revising the paper, we have more directly addressed the similarity between the two methods.

Comment #5: Page 22 Table 5 (but also Table 3): can you try to explain why correlations of WNGF results with the backbone and threshold network is lower for “representative” nodes compared to the other brokerage roles?

We thank the reviewer for the insightful question! After carefully looking at the reason behind this difference in correlation levels in the organization network, we have found the main reason to be the high number of nodes not or rarely becoming representatives. Furthermore, because few nodes take up the representative role, infrequently given advice can already further the knowledge flow between groups in different location. However, both the backbone and threshold results are underestimating the representative roles of the nodes compared to the WNGF results. As seen in the figure below, the largest representative role a node (node 67) takes in the dichotomised network is 0.3, while the representative role of the same node is 0.8 in the WNGF measure. The largest differences between the backbone network and WNGF are for nodes 14,27,48,73 and 67, however there is no common feature of these nodes that would easily explain the lower correlation levels in the case of the representative role.

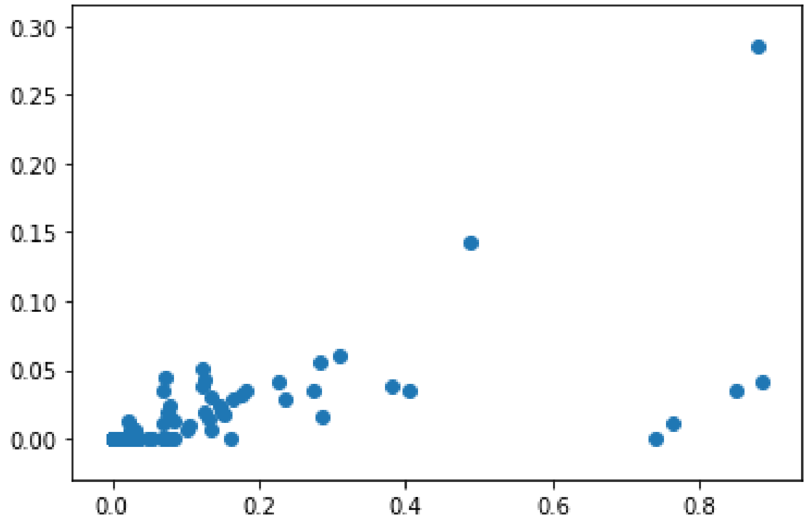

Figure 1. The representative role in case of the WNGF measure vs backbone network

Comment #6: Page 6 “For example, in the context of the US Health policy domain’s communication network (11) by absorbing knowledge from another group and passing it to the other members of their own group;”: move this sentence to a footnote in order to avoid the break of the list.

Unfortunately, *PLOS ONE* does not allow footnotes. However, to keep the flow of the text, we have removed this information.

Comment #7: Check the citation style: it sometimes changes (for instance in page 7 “e.g.: Blau 1986…”). Moreover in page 7 there is a “Fernandez et al. 2000, 2000”.

We agree with the reviewer and apologise for the several mistakes identified. We have now standardized the references in line with the journal’s style.

Comment #8: Page 21 Figure 1: node label color is almost indistinguishable. Please modify the colors.

We have implemented this change on the figure.

Comment #9: Section 4 is a bit lengthy with some repetitions. It could be a bit shortened.

We thank the reviewer for this practical input, we have shortened and revised Section 4.

Comment #10: There are many typos.

We agree with the reviewer and apologise for the several mistakes identified. We have substantially improved the readability of the manuscript

Reviewer #2:

We would like to thank the reviewer for the insightful comments. We took them into serious consideration and undoubtedly they helped us improve the manuscript.

Comment #1: The authors report a proposal to extend brokerage network measures in presence of weighted directed network data. Two procedures for normalising and dichotomising network data have been also adopted. Real data examples are considered to show the usefulness of the proposal. The manuscript is of interest for network analysts but some major revisions are required to improve the readibility and to report the statistical analysis in a more appropriate and rigorous way.

We are very happy to see that the reviewer thinks a weighted GF measure would be of interest to the network community and took the criticism seriously in order to improve the readability and rigour of the analysis.

Comment #2: Abstract - Improve the structure by specifying the added value of the proposed approach to deal with weighted directed networks and the main findings.

We thank the reviewer for highlighting the shortcomings of the abstract, we have amended it to include more details on the value added and the main findings regarding the relevance of the WNGF measure.

Comment #3: Introduction - Improve the readibility of the contribution by better specify the main aim and justify the specific normalisation and dichotomisation procedures adopted among the ones proposed in the literature;

We thank the reviewer for the suggestions. We have improved the introduction to discuss in more detail the main aims and methods. However, as the introduction is quite extensive, we have decided to discuss the choices for the cut-off points further in the methods section.

Comment #4: Introduction - try to avoid repetitive sentences/words by rephrasing the theoretical framework (1.1) and the application fields (1.2) of brokerage measures;

We have now extensively adapted the manuscript accordingly to reduce repetitions and improve structure, flow and understanding.

Comment #5: Section 2 - It could be better to introduce first the methodological approach proposed for the weighted version of the brokerage measures, and then to discuss the different procedures introduced for network data normalisation and dichotomisation.

We have implemented this change in the paper.

Comment #6: Section 2 - Among others, see also the threshold dichotomisation procedure discussed in Giordano and Primerano (2018), and the backbone procedure adopted in Genova et al. (2021) and the references therein.

We have extended the literature review by further discussing threshold and backbone procedures and we have included the suggested references.

Comment #7: Section 2.2 Check Equations 1, 2 and Table 1 in order to be more stable and rigorous adopting the same superscript for the broker node (i or j). In addition, more details are required to

clarify the normalisation version of the GF brokerage roles described in the last column of Table 1 and in the Equation 2.

We thank the reviewer for pointing this out. We have added further details on how to interpret the normalization equations and corrected the mistakes found regarding the superscripts in the description column. We have also added node names to the visualisations, that highlights that the nodes are labelled q, r and s, where node r is always the broker. The i, j and k superscripts are for group membership. Therefore, in the last column when the denominator normalization is described, n_b^i calculates how many different possible brokerage paths any node in group i can take given the brokerage type b.

Comment #8: Section 3 Illustrative examples (Data) - Better clarify the added value in using WNGF measures given that the authors declare "We show that the WNGF measure produces similar results to that of the dichotomized networks but with more in-depth understanding of the role all nodes play." (page 19, 287-288)

We thank the reviewer for their insightful comment. We have clarified this in the abstract as well as in the main body of the paper. We have explained how the fact that the two methods bring consistent results is reassuring and shows that both methods are valid. However, our method has three additional advantages: 1. information retention; 2. It uses data *as it is*, freeing the researcher from assumptions and discretionary choices which may be difficult to justify; 3. It allows for more granularity when the role of less connected nodes is considered.

Comment #10: 3.1 Better describe Matrix Z for EUREGIO dataset and Table 2.

We have further clarified through examples Matrix Z and Table 2 in the manuscript.

Comment #11: 3.1.1. Report more details along the text about the results inserted in Appendix A (page 17, lines 342)

We thank the reviewer for pointing out this shortcoming and have now complemented the text with further details regarding the Supplementary Information.

Comment #12: 3.2 Improve the description of network information and the network visualisation of Figure 2.

We have now updated the network visualisation and added a clarifying sentence to improve the description.

Comment #13: Repetitive sentences are reported at page 20, from line 407 to 422 (see the same sentences at page 19, lines 390-406).

We apologise for this mistake and have now fixed it in the manuscript.

Comment #14: Conclusions - It is not clear the comments regarding computational social sciences and digital data for the scope of the present contribution.

We thank the reviewer for the comment, after reassessing the conclusion we have decided to remove this section.

Comment #15: References - Check all references along the text in line with the journal's style. Different styles are used to cite references (numbers or authors' last name and papers' publication year).

We agree with the reviewer and apologise for the several mistakes identified. We have now standardized the references in line with the journal’s style.

Comment #16: Please clarify the following sentences.

We thank the reviewer for their detailed attention in pointing out these confusing sentences. We have clarified them in the revised manuscript.

Comment #17: Some sentences are repetitive along the text (e.g., nuanced information, nuanced features, nuanced results, nuanced analysis, etc.) and the contribution could be shorten.

We have implemented this change in the paper.

Comment #18: It is suggested to rephrase the titles of the sections according to the main topics (Introduction, Theoretical background, Methodology, Data description and results, Discussion and Conclusions).

We thank the reviewer for this practical input, we have implemented this change in the manuscript.

Comment #19: It is suggested to refine the use of English.

We agree with the reviewer and apologise for the several mistakes identified. We have substantially improved the readability of the manuscript.
